# Dermatologic manifestations of infective endocarditis*

**DOI:** 10.1590/abd1806-4841.20164718

**Published:** 2016

**Authors:** Rafael Tomaz Gomes, Larissa Rezende Tiberto, Viviane Nardin Monte Bello, Margarete Aparecida Jacometo Lima, Gisele Alborghetti Nai, Marilda Aparecida Milanez Morgado de Abreu

**Affiliations:** 1 Universidade do Oeste Paulista (UNOESTE) – Presidente Prudente (SP), Brazil

**Keywords:** Dermatology, Diagnosis, Endocarditis

## Abstract

Despite advances in diagnosis and treatment, infective endocarditis still shows considerable morbidity and mortality rates. The dermatological examination in patients with suspected infective endocarditis may prove very useful, as it might reveal suggestive abnormalities of this disease, such as Osler’s nodes and Janeway lesions. Osler’s nodes are painful, purple nodular lesions, usually found on the tips of fingers and toes. Janeway lesions, in turn, are painless erythematous macules that usually affect palms and soles. We report a case of infective endocarditis and highlight the importance of skin examination as a very important element in the presumptive diagnosis of infective endocarditis.

## INTRODUCTION

Infective endocarditis (IE) is the infection of endocardial structures, including the heart valves and the endocardial wall. IE diagnosis is based on clinical, microbiological, and echocardiographic findings. Antibiotics should be administrated according to the isolated microorganism.^1-3^

The highest rates of IE cases occur in patients with prosthetic heart valves, intracardiac devices, unrepaired cyanotic congenital heart disease, prior history of IE, rheumatic fever, and intravenous drug use.^4^ However, about 50% of IE cases develop in patients with no prior valvular heart diseases. The main etiological agents are *Staphylococcus and Streptococcus*.^1,2^ A skin gateway might be found in 20% of IE cases.^5^

Although dermatological examination plays a crucial role in the evaluation of patients with IE, few studies have described skin manifestations of the disease and its importance for the diagnostic approach to IE. This paper presents a case of IE and discusses the main dermatological findings that patients with this condition might have.

## CASE REPORT

A 42-year-old male patient was seen the emergency room in February 2015 with fever, diffuse abdominal pain, and weight loss, last measured three months prior. He also complained of very poor general condition and weakness. The patient was a chronic alcoholic and non-intravenous drug user. He had been previously hospitalized due to acute pancreatitis in August 2014 and January 2015.

On general physical examination, the patient presented with regular general condition, but was emaciated and pale, with a holosystolic murmur in the mitral valve and edema in both legs. He reported a diffusely painful abdomen, but no palpable masses.

**Figure 1:**
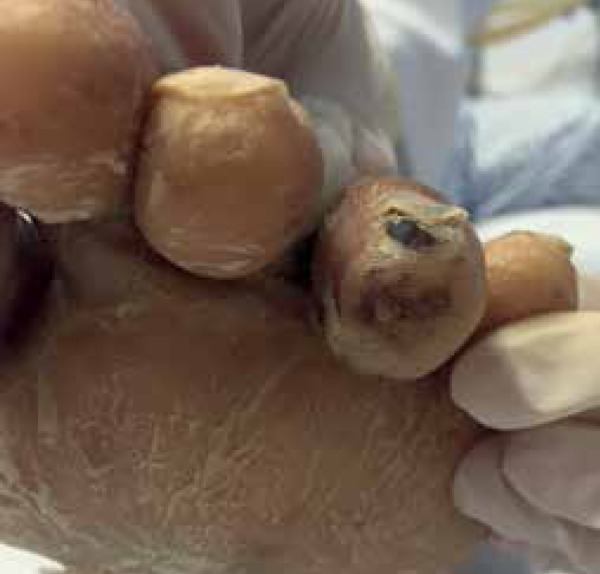
Purple macule on the tip of the fourth left toe and scaly sole

Dermatological examination detected painless purple macules on the tip of the fourth left toe, with absence of superficial necrosis, measuring 4.0 x 5.0 mm (Figure 1), and desquamation of the soles.

The patient was admitted to the hospital and underwent lab tests, which showed the following changes: normochromic normocytic anemia, leukocytosis with absence of left shift, thrombocytosis, hypomagnesemia, increased levels of gamma-glutamyl-transferase, and conjugated hyperbilirubinemia.

The following imaging tests were performed: a) transesophageal echocardiography: mitral valve prolapse with significant failure and imaging suggesting vegetation; b) abdomen and pelvis computed tomography (CT): expansive formation in the head of the pancreas with dilation of intrahepatic or extrahepatic bile duct, probably caused by neoplasia; c) chest CT scan: oversized paratracheal lymph node with contrast uptake heterogeneity; d) head CT scan: hyperdense areas compatible with intracranial hemorrhage, with vasogenic cerebral edema, suggesting metastasis.

**Figure 2:**
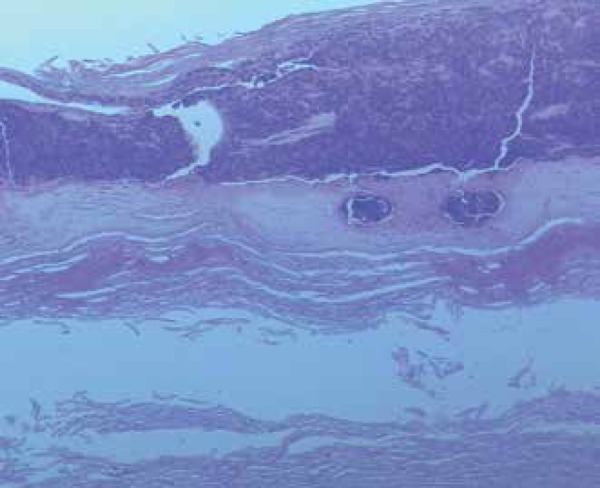
Skin photomicroscopy showing intracorneal pustule. Hematoxylin and eosin, 100x magnification

Blood cultures were positive for *Staphylococcus aureus* in two peripheral blood samples. Skin biopsy (fourh left toe) revealed accumulation of intact and degenerated neutrophils in the dermis and multinucleate giant cells with lymphocytic infiltrate and rare eosinophils, suggesting microabscess (Figure 2).

The following diagnoses were raised chronic alcohol abuse, presumed metastatic adenocarcinoma of the head of the pancreas, and IE with dermatologic manifestations.

The patient’s respiratory pattern eventually worsened, requiring ventilatory support and sedation in a semi-intensive care unit. The skin lesions disappeared after 50 days of antibiotic therapy, as well as the mitral valve vegetation. The patient died during oncological follow-up due to adenocarcinoma of the head of the pancreas.

## DISCUSSION

Despite the importance of skin examination in patients with IE, very few studies describe the major skin-related findings for this disease.

The main IE dermatologic manifestations are Osler’s nodes and Janeway lesions. Such rare signs are found only in 5-15% of IE patients. The prevalence rates may be underestimated due to the lack of a systematic approach to dermatological examination in IE patients.^5,6^

The pathogenesis of Osler’s nodes and Janeway lesions remains controversial. While Osler’s nodes have been traditionally associated with subacute IE, Janeway lesions are usually found in the acute forms of this disease.^7^

Osler’s nodes are painful, purple nodular lesions, usually found on the tips of fingers and toes. However, they can also be found on the thenar and hypothenar eminences and the lateral side of the fingers.^5^ Local pain usually precedes the appearance of lesions by a few hours. The nodes may last from a few hours to several days, and leave no sequelae.^7^

Janeway lesions, on the other hand, are painless purple or brown erythematous macular lesions that usually affect the palms, soles, and fingers. They are sometimes purple or bleeding. They may last days or weeks, and tend to disappear with the resolution of the IE.^5^

Histological findings of both lesions include septic micro-emboli with dermal microabscess formation. Leukocytoclastic vasculitis has also been reported.^5,7,8^ The culture may eventually show a growth of the etiological agent of the IE microorganism.^5,8^

It is believed that Osler’s nodes and Janeway lesions are manifestations of the same pathological process.^7^ The clinical differentiation proposed by some authors is based on clinical features and symptoms of the lesions: painful nodes for the former, and painless macular lesions for the latter.^6,9^

Differential diagnoses include vasculitis, purpura antiphospholipid syndrome, connective tissue diseases, thromboangiitis obliterans, hyperviscosity syndrome, drug eruptions, and gonococcal and meningococcal bacteremia.

We report the case of a patient with IE, with dermatologic manifestation suggesting Janeway lesions. Clinical presentation and histopathological findings are consistent with other cases described in the literature.

Results demonstrate the importance of recognizing the dermatological manifestations of IE. Besides, an early diagnosis is crucial, due to the high morbidity and mortality rates. The identification of Osler’s nodes and Janeway lesions may prove very useful to help a clinician in the presumptive diagnosis of IE.
